# A population study comparing screening performance of prototypes for depression and anxiety with standard scales

**DOI:** 10.1186/1471-2288-11-154

**Published:** 2011-11-22

**Authors:** Helen Christensen, Philip J Batterham, Janie Busby Grant, Kathleen M Griffiths, Andrew J Mackinnon

**Affiliations:** 1 Centre for Mental Health Research, The Australian National University, Canberra, Australia; 2 Centre for Applied Psychology, University of Canberra, Canberra, Australia; 3 Orygen Research Centre, The University of Melbourne, Melbourne, Australia

## Abstract

**Background:**

Screening instruments for mental disorders need to be short, engaging, and valid. Current screening instruments are usually questionnaire-based and may be opaque to the user. A prototype approach where individuals identify with a description of an individual with typical symptoms of depression, anxiety, social phobia or panic may be a shorter, faster and more acceptable method for screening. The aim of the study was to evaluate the accuracy of four new prototype screeners for predicting depression and anxiety disorders and to compare their performance with existing scales.

**Methods:**

Short and ultra-short prototypes were developed for Major Depressive Disorder (MDD), Generalised Anxiety Disorder (GAD), Panic Disorder (PD) and Social Phobia (SP). Prototypes were compared to typical short and ultra-short self-report screening scales, such as the Centre for Epidemiology Scale, CES-D and the GAD-7, and their short forms. The Mini International Neuropsychiatric Interview (MINI) version 6 [1] was used as the gold standard for obtaining clinical criteria through a telephone interview. From a population sample, 225 individuals who endorsed a prototype and 101 who did not were administered the MINI. Receiver operating characteristic (ROC) curves were plotted for the short and ultra short prototypes and for the short and ultra short screening scales.

**Results:**

The study found that the rates of endorsement of the prototypes were commensurate with prevalence estimates. The short-form and ultra short scales outperformed the short and ultra short prototypes for every disorder except GAD, where the GAD prototype outperformed the GAD 7.

**Conclusions:**

The findings suggest that people may be able to self-identify generalised anxiety more accurately than depression based on a description of a prototypical case. However, levels of identification were lower than expected. Considerable benefits from this method of screening may ensue if our prototypes can be improved for Major Depressive Disorder, Social Phobia and Panic Disorder.

## Background

Mental health screening tests identify individuals with a high probability of meeting clinical criteria for a current mental disorder or those who are at risk of developing such a disorder. At the population level, screening is important for targeting treatment and prevention [2], particularly if it is coupled with multi-modal intervention programs such as collaborative care [3,4]. Recent meta-analyses show that screening is associated with a "modest increase in the recognition of depression by clinicians" ([5], p. 997). Typical screening tools are questionnaire-based and, ideally short. Most are completed by the individual rather than the clinician. However, even short screening tools when used as a battery to detect a range of multiple conditions can take a long time to complete. Consequently, researchers are keen to reduce the length of even the shortest tools while maintaining or even improving specificity and sensitivity [6].

In addition to being lengthy, screening instruments can be opaque to the user, boring or baffling, and, thus, be unacceptable to patients. The acceptability of screening tests to the public has rarely been examined, although completion rates of only 30-60% in general practice settings [5] suggest that low acceptability may be a potential problem. Moreover, screening tools may not work well because individuals may find it difficult to label emotions or to recognise they have the symptoms portrayed in many screening items. For example, young people have poor mental health literacy [7-9], reduced emotional competency, may lack the "skills to recognise, interpret and share emotional experiences" ([10], p. 14) and do not necessarily share a common understanding of the construct of "depression" which is tapped by certain depression screeners. In adults, little research exists which focuses on how individuals view depression and whether the construct relates to their own symptoms (see [11] and [12], p. 358), but there is some evidence that adults in primary care have difficulty differentiating depression from "reactions to adversity".

In this study, we sought to develop an alternative methodology in the hope that it might provide a superior approach to screening. We defined "superior" to connote a screening tool that retained high sensitivity, had fast administration time, greater transparency and that might be rated as more satisfactory by users (easier to complete and more enjoyable) in comparison to existing screening tools.

A prototype is the most central or typical member of a category [13]. Prototypes can be used to represent the self and others [14-16]. We wondered if prototypes might serve as a useful means to screen for mental disorders. As a first step in the development of a mental health prototype, we canvassed the research literature to see how prototypes have previously been used. Within mental health, prototype descriptions or typical cases of mental disorders have been developed to assist clinicians make diagnoses. This is most clearly demonstrated by publications such as the DSM-IV Casebooks [17]. However, a more sophisticated approach based on clinician's rating of patients' characteristics has been developed to "refine and dimensionalise existing DSM-IV diagnoses for personality disorder" [18]. In this approach, investigators used a 200 item Q-sort process, where characteristics of patients were rated as applicable or not, with items derived from diverse sources including diagnostic criteria and developmental and personality theories. The prototypes that were developed received positive feedback from clinicians in terms of representing natural diagnosis patterns. As part of the validation procedure, clinicians were asked to determine the extent to which a patient matched or resembled each DSM-IV construct on a 5 point scale. In this task, clinicians were guided by the single-sentence summary that introduces each disorder in the DSM-IV manual ([18], p. 815).

Vignettes (or extended prototypes) [19] have been used as stimuli to determine whether the public can correctly label an individual in a vignette as experiencing a mental disorder such as depression or schizophrenia. In these studies, the vignette was used to determine whether the individual could label disorders based on their description, rather than to test whether they could identify the symptoms as similar to ones they might experience themselves. The closest approach to determining whether prototypes might assist people to identify their own symptoms arises from work investigating children's capacity to identify their own health and welfare, where very young children are asked to identify with puppet prototypes [20]. The above discussion indicates that prototypes may useful in assisting clinicians to identify Axis 1 and Axis II disorders, to determine whether members of the public can name psychiatric disorders, and to assist children to self identify symptoms. These findings indicated that the prototypes might also be useful as screening tools for adults.

The aim of the present project was to evaluate the use of a self-administered screening test in which users match their own thoughts and behaviours to prototypical descriptions of individuals who are experiencing a mental disorder. Prototypes were developed against DSM-IV criteria (see Method). In the interests of developing very brief screening, 'ultra short' prototypes were also developed by distilling the contents of the prototype down to one or two sentences. The short and ultra short prototypes were compared to established brief screening tools and to their short forms. The Center for Epidemiological Studies Depression scale (CES-D 20 item, [21]) and a 10-item short-form of this scale [22] were used to assess depression. The Generalised Anxiety Disorder (GAD-7) is a seven item screener for generalised anxiety disorder [23], with a two-item short-form [24]. The Panic Disorder Severity Scale - Self Report (PDSS-SR) is the self-report form [25] of a scale that includes seven descriptive items for measuring the severity of panic disorder [26]. There are currently no self-report panic disorder scales with a short form available, so for the present study, the short form was based just on the first two items which assess severity and distress of panic attacks. The Social Phobia Inventory (SPIN) is a 17-item scale assessing the severity of social phobia [27], with a three item short form called the Mini-SPIN [28]. The accuracy with which the prototypes (short and ultra short) and the screening tests (standard and short form) identified individuals experiencing each disorder was assessed using the Mini International Neuropsychiatric Interview (MINI) version 6 [1] as a gold standard for caseness.

## Methods

### Participants

Fourteen thousand potential participants were selected randomly from the Electoral Roll, sampling from two federal electorates in the northern suburbs of Sydney, Australia. Registration on the Electoral Roll is compulsory in Australia. Males and females aged 18-65 were included in the sample. Surveys were mailed in May 2009, and by the July 2009 cutoff, 2,976 (21.3%) surveys had been returned. This study received ethics approval from the Human Research Ethics Committee at the Australian National University (Protocol 2009/425).

### Measures

#### Demographics

Information was collected on age, gender, educational level, employment and literacy. Education level was based on four items assessing previous and current educational attainment. Employment status was rated as full-time, part-time and looking for full-time work, part-time, casual, unemployed - looking for work or not in the labour force. Literacy was assessed using three items: language spoken at home, confidence in filling out forms, and frequency of needing help to read printed materials.

#### Prototypes

The measures of interest were short prototypes for Major Depressive Disorder, Generalised Anxiety Disorder (GAD), Panic Disorder and Social Phobia. These are common mental health disorders that often require clinical intervention and are targets of mental health prevention. The prototypes are presented in additional file 1: Prototype items. An additional prototype for schizophrenia (also presented) was developed to act as a control, identifying whether respondents endorsed all items equally or could differentiate between disorders. The prototypes were created using the DSM-IV-TR diagnostic criteria. For each disorder, all possible symptoms listed in the criteria were translated into general descriptions that were designed to be comprehensible to the general adult population. The descriptions were composed into a single, easily-readable paragraph, specifying symptoms and durations, and personified using a fictitious character. The paragraphs were 74-104 words each, depending on the number of symptoms that were required. Respondents were asked to rate how similar they were to the character in the prototype. Responses were given on a seven-point Likert scale, with labelled points at 1, "Not like [name] at all", 4 "Like [name]" and 7 "Exactly like [name]". The Coleman Liau Index was 9.37, and the Flesch Kincaid Grade Level was 7.01 [29]. To keep the prototypes relatively brief, it was not possible to incorporate exclusion criteria, number of symptoms required for diagnosis or subtypes for the disorders. For symptoms that may be bidirectional (e.g., weight increase or decrease), the descriptions were stated as generalities (e.g., Tom's weight has changed recently). The prototype characters were kept as non-specific as possible to encourage identification - only a first name (gender) was provided to make the prototype more understandable. Half of the sample was given the female versions of the prototypes, with the other half receiving the male versions.

Ultra short forms of the prototypes for each of the five disorders were also administered. These are presented in additional file 2: Ultra short prototype items. These items were adapted from previous research investigating mental health literacy [19]. For consistency with their use in prior research, the short form prototypes were rated on a five-point Likert scale: "not at all" (1), "a little" (2), "some" (3), "a fair bit" (4) or "a lot" (5).

#### Standardised screening scales

For comparison, standardised scales were also included in the survey corresponding to each of the disorders assessed by the prototypes. These were the CES-D, the GAD-7, Panic Disorder Severity Scale -Self Report and Social Phobia Inventory (SPIN) and the short forms of these. The order of presentation of the scales was counterbalanced, so that half of the respondents received the prototype items followed by the standard scales, while the other half received the standard scales followed by the prototypes.

#### Diagnostic interview

The Mini International Neuropsychiatric Interview (MINI) version 6 [1] was used as the gold standard for obtaining clinical criteria for comparing the sensitivity and specificity of the prototypes to the scales. The MINI is a brief interview that has strong concordance with diagnoses based on the Structured Clinical Interview for DSM-III-R (SCID) or the Composite International Diagnostic Interview (CIDI) [30]. Only the modules assessing depression, social phobia, panic disorder and generalised anxiety disorder (GAD) were administered, corresponding to each of the prototypes being assessed. Exclusion criteria including drug use, medication use and alternative diagnosis (for GAD) were not assessed, to maintain comparability to the prototypes and scales used in the survey.

### Procedure

Surveys included the short and the ultra short versions of the prototypes, the four standard scales and their short forms (CES-D, GAD-7, SPIN and PDSS-SR), questions on background characteristics, and a consent form for clinical interview. These surveys were mailed to the 14,000 potential participants, along with information about the study. A subsample of respondents was then selected for a clinical interview. An algorithm for clinical interview selection was designed prior to the study, aiming to administer clinical interviews with all of the respondents with high scores on the prototypes and some of the participants with low scores according to a weekly quota system. A random sample of respondents who did not identify with any of the prototypes was also selected for interview. Only participants who provided a telephone number and consented to be interviewed were contacted. Participants who identified at any level with the schizophrenia prototype (*n *= 64) were excluded from having a phone interview.

**Figure 1:**
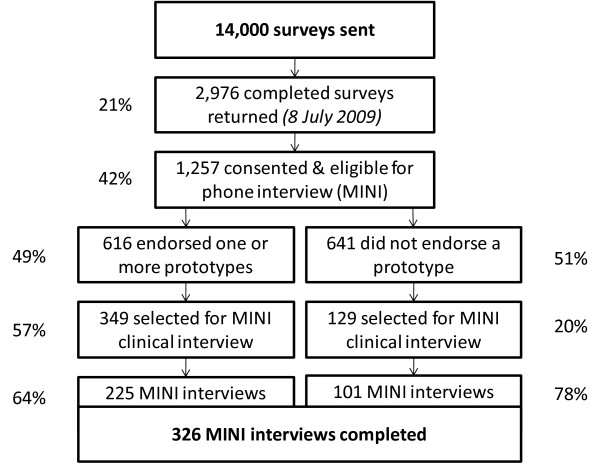
**Sampling procedure and response rates**.

The clinical interviews (MINI) were conducted over the telephone by a team of four clinical psychology postgraduate students and one trainee clinical psychologist, all of whom received three hours of training in the administration of the clinical interview, including a videoconference with the authors of the MINI. From the 2,976 respondents, 1,257 consented and were eligible for a clinical interview. A total of 349 participants who endorsed a prototype and 129 who did not endorse a prototype were selected for clinical interviews. Of those selected, interviews were completed with 225 endorsers (64.5%) and 101 non-endorsers (78.3%). Up to seven call attempts were made in order to contact each of the selected participants, with a two-week window given to make contact after the survey was returned. Clinical interviewers were blinded to the survey responses of the interviewees. The sampling procedure is shown in Figure 1.

### Analyses

Prototype responses were examined across the five disorders, and compared to population rates of caseness. Receiver operating characteristic (ROC) curves were plotted for the prototypes, ultra short prototypes, scales and short-form scales, comparing criteria for caseness on each of these measures to clinical caseness based on the MINI. The critical test of the effectiveness each prototype as a screening tool was to assess whether the area under the short and ultra short prototype ROC curve was as large as the area under the versions of the standard scales and their short forms. Cutoffs for each prototype were established using Youden indices to maximise sensitivity and specificity. The sensitivity and specificity of these cutoffs were compared to the sensitivity and specificity of the scales and short-form scales using their established cutoff scores. Respondents with missing responses on a prototype or scale, or an incomplete module of the MINI were excluded from the comparison for that particular disorder only, resulting in analysis samples of 322 for depression, 324 for GAD, 324 for social phobia and the complete sample of 326 for panic disorder.

## Results

The flow of participants in the trial is shown in Figure 1. The 14,000 people who received the survey were 51.1% female. However, females had a higher response rate to the survey (60.7% of respondents were female). Consequently more clinical interviews were conducted with females (63.5% of interviewees were female), although interviewing rates were not significantly different between female and male respondents [11.4% of female respondents were interviewed vs. 10.3% males, *χ*^2 ^(2) = 2.2, *p *= .332]. The age distributions of respondents to the survey and respondents to the clinical interview were also not significantly different [χ^2 ^(5) = 5.4, *p *= .371]. While efforts were made to select a representative sample, representativeness is not critical for the purposes of comparing multiple measures. The participants who completed a clinical interview were well educated (mean = 14.7 y, *SD *= 2.4 y), with the majority in full-time (*n *= 156, 47.9%) or part-time (*n *= 72, 22.1%) employment. Almost all respondents to the clinical interview spoke exclusively English at home (*n *= 308, 94.5%) and very few relied on assistance for completing forms (*n *= 24; 7.4%) or for reading printed materials (*n *= 15; 4.6%).

**Figure 2:**
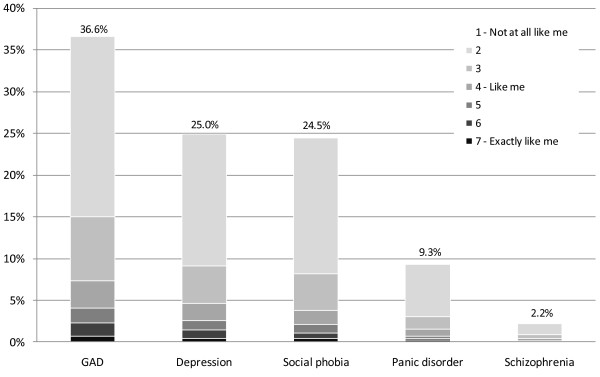
**Rates of prototype endorsement**.

Rates of prototype endorsement across the survey sample (*n *= 2,976) are shown in Figure 2. As category 1 represented no identification with the prototype, this category is not visible in the figure, such that the remainder of respondents (63.4%-97.8%) did not identify at all with the respective prototypes. The percentages listed in the figure represent the percentage of endorsements across all levels from 2-7. A method to assess whether the rates of endorsements was comparable with rates in the general population is to take the rate of clinical caseness (based on the MINI) for each level of prototype endorsement among the 326 clinical interviewees and project these rates across the entire survey sample. Using this method, the base rate of depression in the sample was 6.9%, 5.0% for GAD, 1.7% for social phobia and 1.4% for panic disorder. These base rate estimates, along with the raw prototype endorsement rates, were not dissimilar to the rates of these disorders in the general population: 4.1% for depression, 2.7% for GAD, 4.7% for social phobia and 2.6% for panic disorder [31]. Overall, 51.3% of survey respondents endorsed none of the four prototypes, 20.6% endorsed one prototype at any level (i.e., a rating of 2 or higher), 14.1% endorsed two prototypes and 13.8% endorsed three or four of the prototypes.

**Figure 3:**
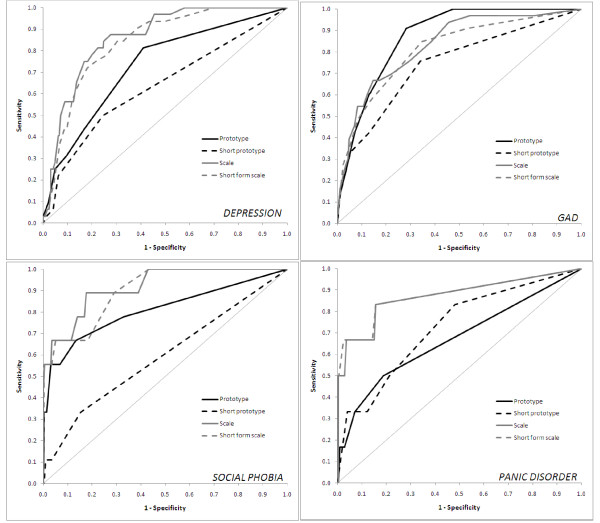
**ROC curves for the prototypes, short-form prototypes, scales and short-form scales for depression, GAD, social phobia and panic disorder**.

The prototypes, ultra short prototypes, scales and short-form scales were compared to MINI criteria using ROC curves, displayed in Figure 3. The MINI identified 33 participants as meeting criteria for GAD and 32 for depression, but only nine for social phobia and six for panic disorder. As is evident from the ROC plots, the scales and short-form scales outperformed the short and ultra short prototypes for every disorder except GAD. Although the AUC confidence intervals of the prototypes overlapped with those of the short-form scales for all of the disorders, the ROC curves suggest that the short form scales performed better than the depression, social phobia and panic prototypes at the assessed cut points. For the GAD prototype, the area under the curve was 0.87, compared to 0.83 for the scale (GAD-7) and 0.81 for the short-form scale (GAD-2). None of the ultra short prototypes was an adequate screener for any of the disorders, as the lower limit of the AUC confidence intervals approached or were below 0.5 and the sensitivity-specificity combinations at the cut points were poor. Results for social phobia and panic disorder may be uncertain, due to the paucity of cases in the sample.

Based on Youden indices to maximise sensitivity and specificity, cutoffs were established for each of the prototypes and ultra short prototypes. For depression and panic disorder, the cut-off that maximised the trade-off between sensitivity and specificity was 2, while for GAD and social phobia it was a score of 3. For the ultra short prototypes, a cut-off of 2 was selected. Sensitivity and specificity for each of these measures, along with the standard scales and short-form standard scales, is shown in Table 1. The cut-offs used for the scales were based on previously established criteria: ≥ 16 for the CES-D [21], ≥ 8 for the CES-D short form [22], ≥ 10 for the GAD-7 [23], ≥ 3 for the GAD-2 [24], ≥ 19 for the SPIN [27], ≥ 6 for the Mini-SPIN [28], ≥ 8 for the PDSS-SR [32], and ≥ 4 for the first two items of the PDSS-SR, corresponding to two or more attacks and moderate or greater distress. The depression prototype had good sensitivity using the cut-off of 2; however, the specificity was poor. Using a cut-off of 3, the GAD prototype had much higher sensitivity with lower (but adequate) specificity compared to both the full standard scale and the short-form scale. The social phobia prototype performed as well as the Mini-SPIN using a cut-off of 3 (or 77.8% sensitivity 67.3% specificity with a cut-off of 2), while the panic disorder prototype was clearly outperformed by the PDSS-SR, although very few respondents met the clinical criteria for these two disorders.

**Table 1:** Sensitivity and specificity (95% confidence intervals) for prototypes and scales at designated cut-offs

Measure	Criterion	Sensitivity (95% CI)	Specificity (95% CI)	AUC (95%CI)
***Depression***				
Full Scale (CES-D)	≥ 16	87.5% (71.0% - 96.5%)	72.4% (66.9% - 77.5%)	0.86 (0.81 - 0.92)
Short form scale (CES-D SF)	≥ 8	84.4% (67.2% - 94.7%)	69.7% (64.0% - 74.9%)	0.84 (0.78 - 0.90)
Depression prototype	≥ 2	81.3% (63.6% - 92.8%)	59.0% (53.1% - 64.7%)	0.73 (0.64 - 0.82)
Depression short prototype	≥ 2	50.0% (31.9% - 68.1%)	75.5% (70.2% - 80.4%)	0.64 (0.54 - 0.73)
***Generalised Anxiety Disorder***				
Full Scale (GAD-7)	≥ 10	60.6% (42.1% - 77.1%)	87.6% (83.3% - 91.1%)	0.83 (0.76 - 0.90)
Short form scale (GAD-2)	≥ 3	57.6% (39.2% - 74.5%)	86.3% (81.8% - 90.0%)	0.81 (0.73 - 0.89)
GAD prototype	≥ 3	90.9% (75.7% - 98.1%)	72.1% (66.5% - 77.2%)	0.87 (0.83 - 0.92)
GAD short prototype	≥ 2	75.8% (57.7% - 88.9%)	66.0% (60.2% - 71.4%)	0.74 (0.65 - 0.83)
***Social phobia***				
Full Scale (SPIN)	≥ 19	88.9% (51.8% - 99.7%)	81.3% (76.6% - 85.5%)	0.92 (0.83 - 1.00)
Short form scale (Mini-SPIN)	≥ 6	66.7% (29.9% - 92.5%)	85.8% (81.4% - 89.4%)	0.90 (0.81 - 0.99)
Social phobia prototype	≥ 3	66.7% (29.9% - 92.5%)	86.7% (82.4% - 90.2%)	0.81 (0.63 - 0.99)
Social phobia short prototype	≥ 2	33.3% (7.5% - 70.1%)	84.8% (80.3% - 88.6%)	0.59 (0.43 - 0.76)
***Panic disorder***				
Full Scale (PDSS-SR)	≥ 8	66.7% (22.3% - 95.7%)	95.6% (92.8% - 97.6%)	0.87 (0.69 - 1.00)
Short form scale (PDSS-SR items 1 & 2)	≥ 4	66.7% (22.3% - 95.7%)	90.3% (86.5% - 93.3%)	0.88 (0.69 - 1.00)
Panic disorder prototype	≥ 2	50.0% (11.8% - 88.2%)	81.3% (76.5% - 85.4%)	0.67 (0.44 - 0.91)
Panic disorder short prototype	≥ 2	83.3% (35.9% - 99.6%)	51.9% (46.3% - 57.5%)	0.73 (0.51 - 0.95)

*Note: *AUC: Area under the curve; CES-D: Center for Epidemiological Studies Depression scale; GAD: Generalised Anxiety Disorder; SPIN: Social Phobia Inventory; PDSS-SR: Panic Disorder Severity Scale - Self Report.

## Discussion

### Summary

The core finding of the present study is that in general, the prototypes did not perform better than the standard screening tools. Indeed, although the scales and prototypes had overlapping confidence intervals, it appeared that the scales and short-form scales outperformed the short and ultra short prototypes for every disorder except GAD. With respect to GAD, the findings suggest that people can self-identify generalised anxiety better than other disorders based on a description of a prototypical case. The reason the GAD prototypes performed as well or better than the screener is unclear. The superiority of the screening scales over the depression prototype may arise because depression symptoms are heterogeneous, or it may be due to the fact that depression modifies people's perceptions of symptoms. It may also be due to the relative positive compared to a negative symptom profile associated with anxiety compared to depression. For example, anxiety is associated with fast heartbeat and sweating. Depression is associated with reduced energy, lower mood, and slower activities. The fact that the GAD prototype was superior or at least as good as the GAD-7 scale suggests that self identification of mental health symptoms is possible. Nevertheless, based on the data collected to date, conventional screening tests are generally more useful than our prototypes for all disorders with the exception of GAD. Because of the small numbers in the social phobia and panic categories, replication of these findings in a larger sample is required.

The study found that the rates of endorsement of the prototypes were commensurate with prevalence estimates, although the rates appeared to be lower for social phobia. Differences in rates between the prototypes and population estimates may be explained by the non-exclusion of other anxiety disorders from GAD caseness in our study, the predictive merits of the prototypes and differing response rates in the community across the disorders. For social phobia, for example, fewer individuals with social phobia may have agreed to the study, or the prototype might have been too strictly limited in symptoms. In addition, the rates of endorsement of the prototypes displayed reasonably good correspondence with existing scales.

### Comparisons to other screening tools

Although most prototypes were not superior to those of the brief screening scales, their performance as screening tools was generally respectable, with sensitivity > 75% at specific cutoffs for the GAD and depression prototypes. Data suggests that standardised questionnaires to detect depression have a median sensitivity of 75% [3]. In general practice settings, researchers have found that single item tests had an overall sensitivity of 31.9% and specificity of 96.0% [33]. Pooled analysis of two or three item tests, found sensitivity of 73.7% and specificity of 74.7%. Like most screening tools, these data suggest that the prototypes may be useful for ruling out depression or anxiety, although not so useful for identifying depression or anxiety levels likely to meet criteria to be a case.

### Limitations of the study

There were insufficient cases of Social Phobia or Panic Disorder to evaluate the protocols for this study - these will need to be further assessed in a future study. The time frame for symptom duration was different across the scales, and this may have affected differences in specificity and sensitivity. We did not measure acceptability of the prototypes or the standard scales. It may be that prototypical screening tools have the advantage that they provide a positive learning experience for the user, facilitate improved self recognition, lead to the intention to seek help, or are associated with higher acceptability. However these factors, along with additional measures of validity and reliability, were not measured in this study, and are the target of ongoing research. Although the response rate of 21% is consistent with other mail-based community surveys, the sample may have been prone to a number of selection biases. The sample was well educated and most were highly literate in English, and it is possible that different outcomes may have arisen if the sample was less well educated. Whether the prototypes might perform better in a less well educated sample requires further research. Nevertheless, the primary aim of the study was to compare the accuracy of responses on two scales (the prototypes vs. standard scales), so representativeness does not diminish this within-person comparison. An additional limitation of the study is that the gold standard employed to detect "caseness" used non-exclusion based diagnosis, and did not attempt differential diagnosis. As such, the use of non-exclusion based diagnosis as the core gold standard will produce different prevalence rates than those based on differential diagnosis. For example, the National Comorbidity Survey reported prevalence estimates with exclusions for the DSM-III-R hierarchical rules [34]. Nevertheless, the methodology used in the present study is commonly applied when large population studies are undertaken. Importantly, however, this methodological limitation does not compromise the aim of the study, which is to compare two methodologies to the same "gold standard" diagnosis.

### Further research

Further research is needed to test the anxiety prototype and to investigate whether the depression prototype might be improved. It is not clear whether prototypes are accurate screening tools for particular individuals, and we are currently investigating predictors, such as previous depression history, to determine for whom the prototypes might be useful. We will also investigate symptoms that are most strongly associated with prototype endorsement and to refine prototype descriptions. We also plan to investigate satisfaction and ease of use [3] for the GAD prototype, and to test the prototypes in clinical populations where their performance requires evaluation.

## Conclusions

We were motivated to develop a new method of screening for a number of reasons, including ease of use and satisfaction for users. Without further refinement, the evidence suggests that, with the exception of GAD, screening for mental health problems at this stage is superior if short screening tools are used.

## Competing interests

The authors declare that they have no competing interests.

## Authors' contributions

HC designed the study and drafted the manuscript, and wrote the grant that supported the research; PJB contributed to the design of the study, managed the study, performed the analyses and drafted parts of the manuscript; JBG contributed to the design of the study, searched the literature and developed the prototypes, managed the study and drafted parts of the manuscript. KMG and AJM assisted in the design of the study and the prototypes, and commented on the manuscript. All authors read and approved of the final version of the manuscript.

## Pre-publication history

The pre-publication history for this paper can be accessed here:

http://www.biomedcentral.com/1471-2288/11/154/prepub

## Supplementary Material

Additional file 1 **Prototype Items**. The prototype measures used in the present study. Click here for file

Additional file 2 **Ultra short prototype items**. The ultra short prototype measures used in the present study. Click here for file
