# Supplementary material for: A population study comparing screening performance of prototypes for depression and anxiety with standard scales
Source: BMC Med Res Methodol. 2011 Nov 22;11:154. doi: 10.1186/1471-2288-11-154 (PMC3235985; doi:10.1186/1471-2288-11-154)
Supplement: Additional file 1 — Prototype Items. The prototype measures used in the present study. [file 1471-2288-11-154-S1.DOCX]

## Prototype items

**Please read the following descriptions carefully.**

**You will be asked how much you are like each person being described.**

1. Jennifer feels sad most of the day, nearly every day. She has lost interest in doing most of the things she used to enjoy doing. Her weight has changed a lot recently, as have her sleeping patterns. Nearly every day she feels very tired and like she has no energy to do anything, but she can’t sit still. She finds it really difficult to concentrate and has a lot of trouble making decisions. Most days she thinks that she is worthless. She has an overwhelming sense of guilt. She repeatedly thinks about death or even about suicide. Things have been like this for more than two weeks now. All of these feelings are so bad that Jennifer has trouble doing ‘normal’ things. She is really upset about the way she has been feeling lately.

Are you:

*_1 2 3 4 5 6 7_*

⬜ ⬜ ⬜ ⬜ ⬜ ⬜ ⬜

Not like Like Exactly

Jennifer Jennifer like

at all Jennifer

2. Most days over the last six months, Nicole has felt very anxious and worried about a lot of different things in her life. She finds it difficult to control how worried she gets. Most days Nicole feels ‘on edge’ and has difficulty concentrating. She is irritable and her muscles feel tense. Although she feels tired all the time, she has difficulty sleeping. These feelings are so bad that Nicole has trouble doing ‘normal’ things. Nicole is really upset about the way she has been feeling lately.

Are you: 
 *_1 2 3 4 5 6 7_*

⬜ ⬜ ⬜ ⬜ ⬜ ⬜ ⬜

Not like Like Exactly

Nicole Nicole like

at all Nicole

3. For the last six months, Kylie has been afraid of particular social situations – usually places where she might have to meet strangers or where other people might look at her. She is worried she might say something or do something really embarrassing. When she has to go into situations like this she feels incredibly anxious, even though she realises that she is overreacting. She tends to avoid these types of events when she can. Together these feelings are so bad that Kylie has trouble doing ‘normal’ things, and this makes it hard for her to meet people. Kylie is really upset about the way she has been feeling lately.

Are you:

*_1 2 3 4 5 6 7_*

⬜ ⬜ ⬜ ⬜ ⬜ ⬜ ⬜

Not like Like Exactly

Kylie Kylie like

at all Kylie

4. Michelle has been having attacks – they come on suddenly – she starts sweating, shaking and her heart starts pounding. She feels as if she can’t breathe and like she’s choking. Her chest hurts and she feels sick and dizzy. Sometimes she feels detached from her body, or her body feels strange or numb, and her body temperature changes. During these attacks she feels she might lose control or die. Having had a couple of these episodes Michelle has now become really worried about having further attacks and what might happen to her if she does. She has changed some of the things she normally does because she is worried about having another attack. She has been like this for over a month now.

Are you:

*_1 2 3 4 5 6 7_*

⬜ ⬜ ⬜ ⬜ ⬜ ⬜ ⬜

Not like Like Exactly

Michelle Michelle like

at all Michelle

5. Rebecca has been hearing voices that other people don’t hear. She feels her thoughts and emotions are being controlled or interfered with. Other people don’t seem to understand what she is talking about and say that she doesn’t make sense. Some people tell her she is behaving rather strangely and they don’t know what she’ll do next. She doesn’t show much emotion or talk as easily or as excitedly as she used to.

Are you:

*_1 2 3 4 5 6 7_*

⬜ ⬜ ⬜ ⬜ ⬜ ⬜ ⬜

Not like Like Exactly

Rebecca Rebecca like

at all Rebecca
