# Supplementary material for: A population study comparing screening performance of prototypes for depression and anxiety with standard scales
Source: BMC Med Res Methodol. 2011 Nov 22;11:154. doi: 10.1186/1471-2288-11-154 (PMC3235985; doi:10.1186/1471-2288-11-154)
Supplement: Additional file 2 — Ultra short prototype items. The ultra short prototype measures used in the present study. [file 1471-2288-11-154-S2.DOCX]

**Ultra short prototype items**

We would like you to consider to what extent you are like these people.

1. ***Melissa*** *finds everyday social contact really uncomfortable. She tries to avoid it as much as she can. She feels afraid that she’ll make a fool of herself.*

Recently to what extent have you felt like Melissa?

⬜*_1_* Not at all

⬜*_2_* A little

⬜*_3_* Some

⬜*_4_* A fair bit

⬜*_5_* A lot

2. ***Karen*** *feels empty and lost. The future looks bleak. She believes she’ll never enjoy anything again.*

Recently to what extent have you felt like Karen?

⬜*_1_* Not at all

⬜*_2_* A little

⬜*_3_* Some

⬜*_4_* A fair bit

⬜*_5_* A lot

3.  *All of a sudden, out of the blue,* ***Belinda*** *felt terrified. Her heart raced and she thought she might be dying. It happened twice this week.*

Recently to what extent have you felt like Belinda?

⬜*_1_* Not at all

⬜*_2_* A little

⬜*_3_* Some

⬜*_4_* A fair bit

⬜*_5_* A lot

4. ***Amanda*** *worries uncontrollably about everything, even little things. She’s restless, on edge and wound-up.*

Recently to what extent have you felt like Amanda?

⬜*_1_* Not at all

⬜*_2_* A little

⬜*_3_* Some

⬜*_4_* A fair bit

⬜*_5_* A lot

5. ***Lisa*** *hears voices and believes things that nobody else thinks are true. She has trouble getting people to understand her and they say she acts strangely.*

Recently to what extent have you felt like Lisa?

⬜*_1_* Not at all

⬜*_2_* A little

⬜*_3_* Some

⬜*_4_* A fair bit

⬜*_5_* A lot

6. Which of **Melissa, Karen, Belinda, Amanda** and **Lisa** (in Questions 1 to 5) are you most like?

⬜*_1_* Melissa

⬜*_2_* Karen

⬜*_3_* Belinda

⬜*_4_* Amanda

⬜*_5_* Lisa

⬜*_9_* None of them
